# Sleep During the COVID-19 Pandemic: Longitudinal Observational Study Combining Multisensor Data With Questionnaires

**DOI:** 10.2196/53389

**Published:** 2024-09-03

**Authors:** Nguyen Luong, Gloria Mark, Juhi Kulshrestha, Talayeh Aledavood

**Affiliations:** 1 Department of Computer Science Aalto University Espoo Finland; 2 Informatics Department University of California, Irvine Irvine, CA United States

**Keywords:** computational social science, digital health, COVID-19, sleep, longitudinal, wearables, surveys, observational study, isolation, sleep patterns, sleep pattern, questionnaires, Finland, fitness trackers, fitness tracker, wearable, sleeping habits, sleeping habit, work from home

## Abstract

**Background:**

The COVID-19 pandemic prompted various containment strategies, such as work-from-home policies and reduced social contact, which significantly altered people’s sleep routines. While previous studies have highlighted the negative impacts of these restrictions on sleep, they often lack a comprehensive perspective that considers other factors, such as seasonal variations and physical activity (PA), which can also influence sleep.

**Objective:**

This study aims to longitudinally examine the detailed changes in sleep patterns among working adults during the COVID-19 pandemic using a combination of repeated questionnaires and high-resolution passive measurements from wearable sensors. We investigate the association between sleep and 5 sets of variables: (1) demographics; (2) sleep-related habits; (3) PA behaviors; and external factors, including (4) pandemic-specific constraints and (5) seasonal variations during the study period.

**Methods:**

We recruited working adults in Finland for a 1-year study (June 2021-June 2022) conducted during the late stage of the COVID-19 pandemic. We collected multisensor data from fitness trackers worn by participants, as well as work and sleep-related measures through monthly questionnaires. Additionally, we used the Stringency Index for Finland at various points in time to estimate the degree of pandemic-related lockdown restrictions during the study period. We applied linear mixed models to examine changes in sleep patterns during this late stage of the pandemic and their association with the 5 sets of variables.

**Results:**

The sleep patterns of 27,350 nights from 112 working adults were analyzed. Stricter pandemic measures were associated with an increase in total sleep time (TST) (β=.003, 95% CI 0.001-0.005; *P*<.001) and a delay in midsleep (MS) (β=.02, 95% CI 0.02-0.03; *P*<.001). Individuals who tend to snooze exhibited greater variability in both TST (β=.15, 95% CI 0.05-0.27; *P*=.006) and MS (β=.17, 95% CI 0.03-0.31; *P*=.01). Occupational differences in sleep pattern were observed, with service staff experiencing longer TST (β=.37, 95% CI 0.14-0.61; *P*=.004) and lower variability in TST (β=–.15, 95% CI –0.27 to –0.05; *P*<.001). Engaging in PA later in the day was associated with longer TST (β=.03, 95% CI 0.02-0.04; *P*<.001) and less variability in TST (β=–.01, 95% CI –0.02 to 0.00; *P*=.02). Higher intradaily variability in rest activity rhythm was associated with shorter TST (β=–.26, 95% CI –0.29 to –0.23; *P*<.001), earlier MS (β=–.29, 95% CI –0.33 to –0.26; *P*<.001), and reduced variability in TST (β=–.16, 95% CI –0.23 to –0.09; *P*<.001).

**Conclusions:**

Our study provided a comprehensive view of the factors affecting sleep patterns during the late stage of the pandemic. As we navigate the future of work after the pandemic, understanding how work arrangements, lifestyle choices, and sleep quality interact will be crucial for optimizing well-being and performance in the workforce.

## Introduction

### Background

Sleep is a crucial component of daily life, closely interconnected with all aspects of our routines and overall well-being, including mental health [1,2], physical health [3], and work performance [4,5]. The COVID-19 pandemic profoundly impacted various aspects of daily life, with sleep patterns being a particularly significant area of concern. However, the effects on sleep were often indirect, resulting from changes in daily routines and lifestyle adjustments rather than being a direct consequence of the virus.

In response to the pandemic, outdoor restrictions limited our exposure to natural daylight, a crucial element for regulating circadian rhythms and sleep patterns [6]. Similarly, mobility restrictions altered daily physical activity (PA) patterns. Additionally, workplace restrictions led to work-from-home policies, which resulted in reduced mobility and flexible working hours. While these changes led to more relaxed work schedules, they also blurred the boundaries between professional and personal life. Notably, factors such as daylight exposure, PA, and work routines—each significantly affected by the pandemic—are well-established influences on sleep health [7,8].

Traditional sleep measurements often rely on self-reported methods, such as the Karolinska [9] or Pittsburgh sleep diary [10]. While these methods are effective for tracking day-to-day sleep over short periods, conducting diary studies over longer intervals is generally not feasible due to the cognitive burden on participants. Nonintrusive measurements using smartphones and fitness trackers have recently emerged as a more viable alternative for capturing sleep data over extended periods. While consumer-grade devices may not precisely detect sleep stages, they have shown promising results for measuring sleep onset, duration, and wake-up time. Assessing sleep with these devices has the advantage of capturing data in people’s natural living environments, unlike sleep laboratories. Additionally, this method is not subject to memory biases that can occur with survey responses and sleep diaries.

The evolution of mobile health (mHealth) technologies has significantly enhanced traditional sleep monitoring methods, particularly through the use of wearable devices. These devices offer a more accessible and less invasive way to monitor sleep patterns, while also deepening our understanding of sleep-related phenomena. For instance, wearable devices have been used to determine individuals’ chronotypes and track their sleep and activity rhythms over extended periods [11,12]. They have also been used to measure sleep alignment between coworkers [13], examine the relationship between sleep and burnout [14], and assess sleep patterns in various populations, including patients with mental disorders [15]. Several studies have confirmed the validity and reliability of wearable devices, demonstrating notable sensitivity compared with the gold-standard polysomnography (PSG). For example, a review of 7 consumer sleep-tracking devices [16] highlighted their high effectiveness in detecting sleep relative to PSG. Similarly, a study [17] evaluated 6 consumer wearable devices and validated their accuracy in assessing sleep timing and duration compared with PSG.

Prior research comparing sleep patterns before and during the pandemic has revealed notable differences. Studies found that following the pandemic’s onset, individuals tended to go to bed later [18], slept for longer durations [19], exhibited reduced variability between weekday and weekend sleep [20,21], and experienced increased sleep disturbances or diminished sleep quality [22]. Various factors have been identified as contributing to these disruptions in sleep routines, including decreased PA [23], social isolation [24], increased use of electronic devices [4], and the shift to working from home [13].

While previous studies have focused on the immediate consequences of lockdowns and restriction policies, less attention has been paid to the long-term effects, particularly during the late stages of the pandemic when restrictions began to relax. This phase is crucial for understanding the residual effects of the pandemic on sleep patterns and how quickly individuals revert to their prepandemic sleep habits. The transition to working from home as the default mode has resulted in a less constrained work-life routine, leading to more flexible sleep-wake schedules. Certain demographics may benefit more from these transitions, such as individuals with more flexible routines (eg, research personnel) or those who tend to snooze their alarms after waking, referred to as “snoozers.” Additionally, occupation is a known factor influencing sleep patterns, with a classic example being the contrast between shift workers and nonshift workers [25,26]. However, less is understood about the differences between various roles within academia, such as researchers with deadline-driven roles and administrative personnel typically following a 9-to-5 schedule. Therefore, a comprehensive, longitudinal analysis of sleep patterns that includes these variables and extends into the late stages of the pandemic is important.

### Objectives

Our study aims to provide a holistic view of how the pandemic has influenced sleep patterns. We evaluate the long-term relationships between sleep patterns, including average and variability in total sleep duration and sleep timing, alongside individuals’ characteristics (demographics, occupation, and PA) and external factors (stringency of restriction policies and seasonal variations). Our research utilizes longitudinal data from fitness trackers and questionnaire responses collected from working adults at a Finnish university. This extensive data set enables us to examine shifts in sleep behavior during the later stages of the COVID-19 pandemic, from June 2021 to June 2022. The study’s timeframe covers a full annual seasonal cycle, which is crucial for analyzing sleep patterns in Finland, where significant seasonal changes and daylight variations occur due to its northern latitude.

## Methods

### Study Data

This work used data from the cor:ona (comparison of rhythms: old vs. new) study [27] as part of a 1-year multimodal data set of working adults.

### Ethics Approval

The study was approved by the Aalto University Research Ethics Committee (approval number D/536/03.04/2021_COR_ONA).

### Participants and Procedures

The cor:ona study recruited 128 full-time employees from a university in Finland for a 1-year investigation into how their daily activities changed during different stages of the COVID-19 pandemic. Throughout the study, participants wore a Polar Ignite fitness tracker (Polar Electro Oy), enabling us to unobtrusively collect various measures related to sleep and PA. In addition, participants completed an initial baseline questionnaire, an exit questionnaire, and a shorter version of the baseline questionnaire each month. The monthly questionnaires asked for information about their daily routines, work, and sleep quality over the past month. The detailed recruitment procedure and participants’ demographics were described in a previous study [27].

### Fitness Tracker Data

#### Sleep Measures

The fitness trackers measured bedtime (defined as the recorded time when a person fell asleep), waketime (defined as the recorded time when a person woke up), and interruption duration (defined as the total time in seconds spent awake between sleep start and end times) for each day. A sleep period was defined as the longest sleep episode for each day. Sleep patterns were measured using 4 metrics: (1) total sleep time (TST), which measured the time a person spent asleep, calculated as the duration from bedtime minus the interruption duration; (2) midsleep (MS), the midpoint between bedtime and waketime, which was used to measure sleep timing and computed as (bedtime + TST)/2. Additionally, we proposed 2 other metrics to measure sleep regularity: (3) TST variability, computed as the SD of TST during weekdays (Sunday night to Thursday night); and (4) MS variability, computed as the SD of MS during weekdays. We focused exclusively on weekdays due to the expected differences between weekday and weekend sleep patterns. The Niimpy behavioral data analysis toolbox [28] was used for extracting sleep measurements.

#### Physical Activity Measures

The fitness tracker recorded the number of steps taken each hour, which were then summed to provide a daily step count. To comprehensively account for daily PA patterns, including their timing and distribution, we introduced 2 additional metrics: midstep and intradaily variability (IV) [29]. These metrics are designed to capture the timing and dispersion of PAs throughout the day. Specifically, midstep represents the hour of the day when half of the total number of steps is achieved, analogous to MS in the context of PA. By contrast, IV quantifies the fragmentation of the activity-rest rhythm and is measured as follows:




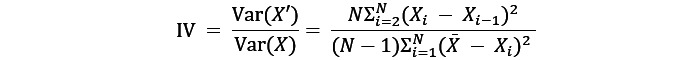




where *N*=24 is the total number of samples within each day; *X_i_* is the *i* measurement sampled at *P*=60-minute interval; and 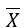 is the average value of all samples in a day. Low IV indicates less fragmented activity-rest rhythm, whereas high IV could imply daytime naps or nighttime awakenings.

#### External Data

Seasonal data were collected from the World Weather Online developer application programming interface [30]. Given the significant variation in day length in Finland during the study (up to 13 hours), day length was used as a proxy for seasonal variables. The choice of day length as a proxy was motivated by Friborg et al [31]. The study compared 2 geographically distinct locations with substantial differences in day length variability: Ghana and Norway. Although no noticeable seasonal effects of day length were observed in Ghanaians, Norwegians showed a delay in both bedtime and waketime during summer weekdays, though sleep duration remained relatively unaffected.

We also utilized the Stringency Index (SI) [32], a composite measure ranging from 0 to 100, to assess daily COVID-19 restriction policies. Higher values on this index indicate more stringent COVID-19 restrictions, including measures such as school and workplace closures, the cancellation of public events, and the enforcement of stay-at-home orders. This index allows for standardized comparisons of policy responses across different countries or regions, as well as changes within the same region over time.

#### Questionnaire Data

Upon entering the study, participants completed a baseline questionnaire that collected basic background information, including age, gender, chronotype, occupation, and origin, among others. Chronotype was assessed using the reduced Morningness-Eveningness Questionnaire (MEQ) [33], with higher scores indicating a morning type and lower scores indicating an evening type. For the origin-related question, participants chose from 3 options: Finland, Europe (excluding Finland), or outside of Europe. Participants indicating they were from Finland were classified as Finnish, while those selecting other options were described as having a “migrant background.” Regarding occupation, participants specified whether they were academic or service staff. The term “academic staff” refers to individuals involved in academic and research activities within the organization, while “service staff” includes those in roles such as human resources and other administrative or support functions. Participants were determined as a snoozer if they answered “yes” to the following question: “Snoozing can be considered as choosing to go back to sleep after an alarm has awakened you intending to wake up later; setting the alarm earlier than when you intend to wake up; or setting multiple alarms with the intent to not wake up on the first alarm. Do you currently consider yourself a snoozer using this definition?,” as adapted from [34].

For the analysis of snoozer characteristics, we used the 2-item Patient Health Questionnaire (PHQ-2) [35] and the short form of the Pittsburgh Sleep Quality Index (PSQI) [10], averaging the values collected from the monthly questionnaires. Additionally, the short form of the Positive and Negative Affect Schedule (PANAS-SF) [36] was used in the initial baseline questionnaire.

### Data Exclusion and Preprocessing

Sleep data were restricted to the period from July 1, 2021, to May 31, 2022. Because of our rolling recruitment process, which started in mid-June 2021 and ended in June 2022, we excluded data from June of both years. This exclusion was necessary because we lacked complete data for these months, and including partial data could have introduced bias. A standard filter, adopted from [37], was applied to remove outliers in TST (TST<3 hours and TST>13 hours). Participants with fewer than 30 recorded nights due to dropout or technical issues were excluded. For gender-related analysis, nonbinary participants (n=1) were excluded to preserve their privacy. To maintain the interpretability of the relationships between sleep patterns and the examined variables, we chose not to normalize the dependent and independent variables.

### Statistical Analysis

We used a logistic regression model to examine factors predicting snoozing behavior. Using snoozing behavior as the dependent variable, and to replicate the findings from [34], we included the same set of independent variables: age, gender, step count, TST, BIG-5 personality traits (openness, conscientiousness, extraversion, agreeableness, and neuroticism), PANAS-SF, PHQ-2, PSQI, and MEQ. To further investigate the potential confounding effects of chronotype (measured by MEQ) on the relationship between personality traits and snoozing behavior, we conducted a Baron and Kenny [38] mediation analysis.

Given the nature of our data set, which included repeated sleep measurements for each participant, we used mixed effects linear models [39] to analyze how sleep patterns and their regularity evolve over time. The models included TST, MS, and the variability of TST and MS as dependent variables. For models with variability of TST and MS as dependent variables, the numerical independent variables were averaged across weekdays. We adopted a sequential modeling strategy, building 3 distinct models for each dependent variable. Model 1 included basic characteristics such as chronotype, age, gender, origin, occupation, and parenting cohabitation status (number of children in the household). Model 2 extended model 1 by adjusting for external factors such as the stringency of restrictions and day length. Finally, model 3 built on model 2 by incorporating PA metrics, including step count, midstep, and IV. This approach allows for the exploration of the unique contributions of each new set of variables beyond those accounted for in the previous model. All models included hierarchical random effects for the study participants to account for repeated measurements. The models are formulated as follows:

Model 1: *Y_ij_* = *β*_0_ + *β*_1_*x_ij_*_1_ + *β*_2_*xij*_2_ +···+ *β*_2_*xij*_7_ + *β*_8_*x_ij_*_8_ + *u_j_* + *ϵ_ij_*

Model 2: *Y_ij_* = *β*_0_ + *β*_1_*x_ij_*_1_ + *β*_2_*xij*_2_ +···+ *β*_9_*xij*_9_ + *β*_10_*x_ij_*_10_ + *u_j_* + *ϵ_ij_*

Model 3: *Y_ij_* = *β*_0_ + *β*_1_*x_ij_*_1_ + *β*_2_*xij*_2_ +···+ *β*_11_*xij*_11_ + *β*_12_*x_ij_*_12_ + *β*_13_*x_ij_*_13_ + *u_j_* + *ϵ_ij_*

where the independent variables are *x_ij_*_1_=age, *x_ij_*_2_=gender, *x_ij_*_3_=number of children, *x_ij_*_4_=origin, *x_ij_*_5_=occupation, *x_ij_*_6_=MEQ, *x_ij_*_7_=snoozer, *x_ij_*_8_=free day, *x_ij_*_9_=Stringency Index, *x_ij_*_10_=day length, *x_ij_*_11_=steps(×1000), *x_ij_*_12_=midstep, and *x_ij_*_13_=step entropy.

95% CIs were reported using bootstrapping. The performance of the model was compared using the likelihood ratio test (LRT) to ensure model parsimony. All statistical analyses were performed using R software (version 3.6.1; R Foundation) [40]. Linear mixed models were tested using the lme4 package [41], and *P* values for these models were calculated using the lmerTest package [42].

## Results

### Data Summary

**Figure 1:**
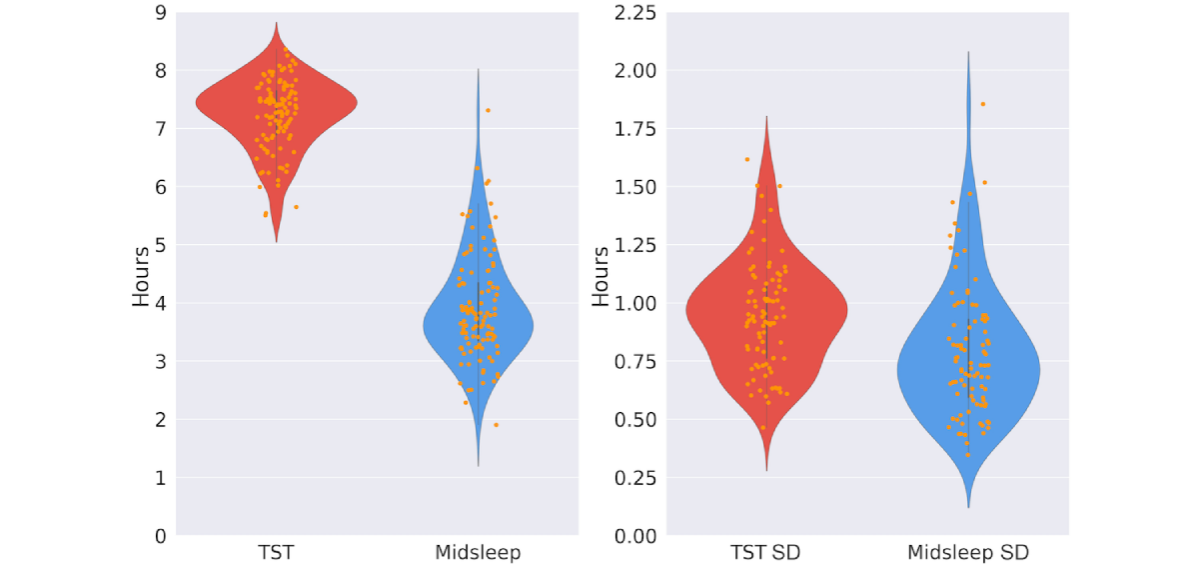
TST, MS, and their SDs of participants included in the analysis. Each dot represents the participant's mean value for the corresponding metrics. MS: midsleep; TST: total sleep time.

**Figure 2:**
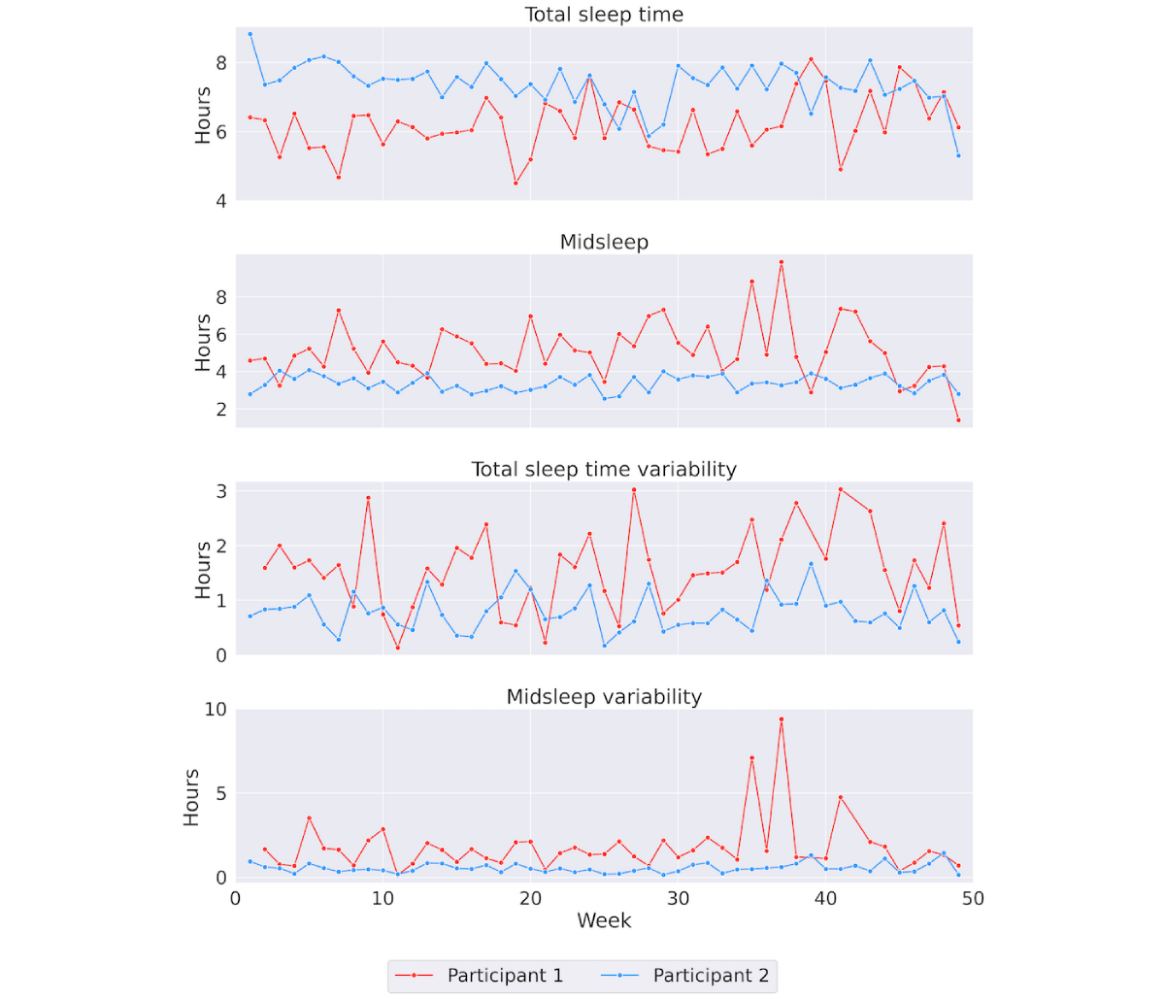
Sleep data over time from two participants. Participant 1 (red line) demonstrates shorter, later, and more variable sleep compared to Participant 2 (blue line).

In total, 112 users and 27,350 nights were included in the TST and MS analyses. The models for the variability of TST and MS used the weekday SD of both measures, which included 3682 observations. The average age of participants was 39.5 (SD 9.9) years. Of these 112 participants, 49 were academic staff and 63 were service staff. Figure 1 presents the average values of the 4 sleep metrics—TST, MS, and their corresponding SDs—for each participant included in the analysis. Figure 2 illustrates the sleep patterns over time for 2 participants: 1 with low variability and 1 with high variability in their sleep patterns.

### Total Sleep Time

We begin by investigating the factors that influence TST using the 3 linear mixed models described earlier. Table 1 presents the results of these models predicting TST. For improved interpretability, the rate of change in TST is expressed as the estimate of the predictors multiplied by 60 minutes. In the full model (model 3), an increase in age by 1 year was associated with a 1.2-minute decrease in TST (95% CI –1.8 to –0.6; *P*=.008). Regarding gender, males were found to sleep 20.4 minutes less than females (95% CI –33.0 to –7.8; *P*<.001). Comparing occupations, service staff were found to sleep 22.2 minutes more than academic staff (95% CI 8.4-36.6; *P*=.004). A detailed monthly breakdown of the variations in sleep pattern measurements across different occupations is provided in Multimedia Appendix 1. After adjusting for day length and the SI, an additional hour of day length was associated with a 0.60-minute decrease in TST (95% CI –0.72 to –0.36; *P*<.001). Conversely, a 1-point increase in the SI offset this decrease by 0.18 minutes (95% CI 0.06-0.30; *P*<.001). In the full model, which included PA, a 1-unit increase in IV was associated with a 15.6-minute decrease in TST (95% CI –17.5 to –13.8; *P*<.001). Moreover, an additional hour in midstep was associated with a 1.8-minute increase in TST (95% CI 1.2-2.4; *P*<.001).

**Table 1:** Estimates of fixed effects from the linear mixed effects model predicting TST^a^.

Predictors	Model 1^b^				Model 2^c^				Model 3^d^		
	Estimated	CI	*P* value^e^	Estimated		CI	*P* value^e^	Estimated		CI	*P* value^e^
Age	–0.02	–0.03 to –0.01	*.002^f^*	–0.02		–0.03 to –0.01	*.002^f^*	–0.02		–0.03 to –0.01	*.008^f^*
Gender (male)	–0.34	–0.55 to –0.14	*<.001^g^*	–0.34		–0.55 to –0.14	*<.001^g^*	–0.34		–0.55 to –0.13	*<.001* ^g^
Number of children	0.04	–0.07 to 0.15	.51	0.04		–0.07 to 0.15	.51	0.03		–0.07 to 0.14	.56
Origin (migrant background)	0.01	–0.27 to 0.27	.97	0.01		–0.27 to 0.27	.97	0.01		–0.24 to 0.24	.93
Occupation (service)	0.36	0.11 to 0.60	*.01^h^*	0.36		0.11 to 0.60	*.01^h^*	0.37		0.14 to 0.61	*.004^f^*
MEQ	–0.01	–0.04 to 0.02	.59	–0.01		–0.04 to 0.02	.60	0.30		–0.20 to 0.22	.79
Snoozer (Yes)	–0.2	–0.44 to 0.05	.11	–0.2		–0.44 to 0.05	.11	–0.18		–0.44 to 0.05	.14
Free day (Yes)	0.10	0.07 to 0.13	*<.001^g^*	0.10		0.07 to 0.13	*<.001^g^*	0.08		0.06 to 0.11	*<.001^g^*
Stringency Index	—^i^	—	—	0.005		0.003 to 0.007	*<.001^g^*	0.003		0.001 to 0.005	*<.001^g^*
Day length	—	—	—	–0.01		–0.01 to –0.01	*<.001^g^*	–0.01		–0.012 to –0.006	*<.001^g^*
Steps (×1000)	—	—	—	—		—	—	–0.01		–0.01 to 0.01	<.001^g^
Midsteps	—	—	—	—		—	—	0.03		0.02 to 0.04	<*.001*^g^
Intradaily variability	—	—	—	—		—	—	–0.26		–0.29 to –0.23	*<.001^g^*

^a^The σ^2^ values for models 1-3 were 1.13, 1.13, and 1.1, respectively. The intraclass correlation coefficient values for models 1-3 were 0.19, 0.19, and 0.19, respectively. The marginal *R*^2^/conditional *R*^2^ values for models 1-3 were 0.055/0.234, 0.057/0.235, and 0.069/0.245, respectively. The Akaike information criterion values for models 1-3 were 81,208.16, 81,154.98, and 80,783.26, respectively.

^b^Includes demographic and occupational variables.

^c^Includes model 1 + restriction and seasonal factors.

^d^Includes model 2 + physical activity influences.

^e^Italicized values denote significance.

^f^*P*<.01.

^g^*P*<.001.

^h^*P*<.05.

^i^Not available.

The marginal *R*^2^ values represent the proportion of variance explained by the fixed effects, while the conditional *R*^2^ values indicate the proportion of variance accounted for by both fixed and random effects. The increase in both *R*^2^ values suggests that more complex models, particularly model 3, explained a greater proportion of the variance in the dependent variable. The LRT between models 1 and 2 indicated that model 2 was a significantly better fit (*χ*^2^_2_=57.17; *P*<.001). Additionally, the LRT between models 2 and 3 showed that model 3 provided a significantly improved fit (*χ*^2^_3_=377.72; *P*<.001). The performance of the full model (model 3) was further supported by the Akaike information criterion (AIC), which was lowest for model 3 (AIC 80,783.26), indicating that it offered the most optimal fit for the data.

### Midsleep

Using the same approach, we developed 3 linear mixed models to assess the associations between the same set of predictors and MS. The results are presented in Table 2. To enhance interpretability, the rate of change in MS is measured as the estimate of the predictors multiplied by 60 minutes. Across all 3 models, chronotype (MEQ) (*P*<.001) and sleep on a free day (*P*<.001) consistently emerged as significant factors. In the full model (model 3), a 1-point increase in the MEQ was associated with an 8.4-minute decrease in MS (95% CI –10.8 to –5.4; *P*<.001). Sleep on a free day occurred 11.4 minutes later (95% CI 9.6-12.6; *P*<.001) compared with a workday. After adjusting for season and restriction policies, MS was delayed by 0.6 minutes (95% CI 0.6-1.2; *P*<.001) for each additional hour of day length. A 1-point increase in the SI was associated with a 1.2-minute increase in MS (95% CI 1.2-1.8; *P*<.001). In the full model, which included PA variables, a 1-unit increase in IV was linked to a 17.4-minute earlier MS (95% CI –19.8 to –15.6; *P*<.001). Similarly, an increase in step count was associated with a 0.6-minute earlier MS (95% CI –1.2 to 0.0; *P*=.04).

The LRT between models 1 and 2 indicated that model 2 was a better fit (*χ*^2^_2_=443.70; *P*<.001). Additionally, the LRT between models 2 and 3 showed that model 3 provided a significantly improved fit (*χ*^2^_3_=291.63; *P*<.001). The AIC value for model 3 was also the lowest (AIC 86,315.145), indicating that it provided the best fit for the data.

**Table 2:** Estimates of fixed effects from the linear mixed effects models predicting MS^a^.

Predictors	Model 1^b^				Model 2^c^				Model 3^d^		
	Estimated	CI	*P* value^e^	Estimated		CI	*P* value^e^	Estimated		CI	*P* value^e^
Age	–0.01	–0.02 to 0.01	.38	–0.01		–0.02 to 0.01	.46	–0.006		–0.02 to 0.01	.56
Gender (male)	0.13	–0.16 to 0.41	.39	0.12		–0.17 to 0.40	.43	0.11		–0.19 to 0.38	.49
Number of children	–0.1^f^	–0.33 to –0.02	*.02^f^*	–0.18		–0.33 to –0.02	*.02^f^*	–0.14		–0.30 to 0.02	.08
Origin (migrant background)	0.19	–0.18 to 0.55	.32	0.18		–0.20 to 0.53	.34	0.09		–0.29 to 0.02	.63
Occupation (service)	–0.17	–0.51 to 0.17	.33	–0.18		–0.52 to 0.15	.29	–0.21		–0.55 to 0.14	.23
MEQ	–0.14	–0.18 to –0.09	*<.001^g^*	–0.14		–0.18 to –0.09	*<.001^g^*	–0.14		–0.18 to –0.09	*<.001^g^*
Snoozer (Yes)	0.27	–0.06 to 0.61	.09	0.29		–0.04 to 0.63	.08	0.32		–0.05 to 0.66	.08
Free day (Yes)	0.21	0.18 to 0.24	*<.001^g^*	0.21		0.18 to 0.24	*<.001^g^*	0.19		0.16 to 0.21	*<.001^g^*
Stringency Index	—^h^	—	—	0.02		0.02 to 0.03	*<.001^g^*	0.02		0.02 to 0.03	*<.001^g^*
Day length	—	—	—	0.00		0.00 to 0.01	*.048^f^*	0.01		0.00 to 0.01	*.002^i^*
Steps (×1000)	—	—	—	—		—	—	–0.01		–0.02 to –0.01	*<.001^g^*
Midsteps	—	—	—	—		—	—	0.00		–0.00 to 0.01	.43
Intradaily variability	—	—	—	—		—	—	–0.29		–0.33 to –0.26	<.*001*^f^

^a^The σ^2^ values for models 1-3 were 1.38, 1.36, and 1.36, respectively. The intraclass correlation coefficient values for models 1-3 were 0.26, 0.26, and 0.27, respectively. The marginal *R*^2^/conditional *R*^2^ values for models 1-3 were 0.168/0.389, 0.178/0.400, and 0.179/0.400, respectively. The Akaike information criterion values for models 1-3 were 86,990.188, 86,573.060, and 86,315.145, respectively.

^b^Includes demographic and occupational variables.

^c^Includes model 1 + restriction and seasonal factors.

^d^Includes model 2 + physical activity influences.

^e^Italicized values denote significance.

^f^*P*<.05.

^g^*P*<.001.

^h^Not available.

^i^*P*<.01.

### Total Sleep Time Variability

Table 3 presents the factors predicting the variability in TST. Across the 3 models, age (*P*=.01), number of children (*P*=.03), occupation (*P*<.001), and snoozing behavior (*P*=.006) emerged as significant factors. In the final model (model 3), each additional year of age was associated with a 0.01-unit increase in TST variability (95% CI 0.00-0.01; *P*=.01). Notably, participants with snoozing habits exhibited higher TST variability, increasing by 0.15 units (95% CI 0.05-0.27; *P*=.006). Each additional child was associated with a 0.06-unit reduction in TST variability (95% CI –0.11 to –0.00; *P*=.03). Service staff also demonstrated lower TST variability, with a reduction of 0.15 units compared with academic staff (95% CI –0.27 to –0.05; *P*<.001). When accounting for PA, a decrease of 1 hour in midsteps was correlated with a 0.01-unit increase in TST variability (95% CI –0.02 to –0.00; *P*=.03), while a 1-unit increase in IV was associated with a 0.16-unit decrease in TST variability (95% CI –0.23 to –0.09; *P*=.03). The LRT indicated that model 2 did not provide an improvement over the baseline model (*χ*^2^_2_=4.78; *P*=.09). However, model 3 demonstrated better performance compared with the baseline model (*χ*^2^_5_=31.95; *P*<.001).

**Table 3:** Estimates of fixed effects from the linear mixed effects model predicting TST variability^a^.

Predictors	Model 1^b^				Model 2^c^				Model 3^d^			
	Estimated	CI	*P* value^e^	Estimated		CI	*P* value^e^	Estimated		CI	*P* value^e^	
Age	0.01	0.00 to 0.01	*.01^f^*	0.01		0.00 to 0.01	*.01^f^*	0.01		0.00 to 0.01	*.01^f^*	
Gender (male)	0.11	0.01 to 0.21	*.038^g^*	0.11		0.01 to 0.21	*.03^g^*	0.10		0.00 to 0.21	.06	
Number of children	–0.05	–0.11 to 0.00	.056	–0.05		–0.11 to 0.00	.052	–0.06		–0.11 to –0.00	*.01^g^*	
Origin (migrant background)	–0.03	–0.15 to 0.10	.56	–0.03		–0.15 to 0.09	.54	–0.03		–0.15 to 0.08	.56	
Occupation (service)	–0.17	–0.28 to –0.05	*.004^g^*	–0.17		–0.28 to –0.05	*.004^g^*	–0.15		–0.27 to –0.05	*<.001^h^*	
MEQ	0	–0.01 to 0.02	.55	0		–0.01 to 0.02	.57	0		–0.01 to 0.02	.60	
Snoozer (yes)	0.18	0.07 to 0.30	*.002* ^g^	0.18		0.07 to 0.30	*.002^f^*	0.15		0.05 to 0.27	*.006^f^*	
Daylength	—^i^	—	—	0		–0.00 to 0.01	.16	0		–0.00 to 0.01	.08	
Stringency Index	—	—	—	0		–0.00 to 0.00	.10	0		–0.00 to 0.01	.17	
Steps (×1000)	—	—	—	—		—	—	–0.01		–0.01 to 0.00	.007^g^	
Midsteps	—	—	—	—		—	—	–0.01		–0.02 to –0.00	*.02^f^*	
Intradaily variability	—	—	—	—		—	—	–0.16		–0.23 to –0.09	*.001^h^*	

^a^The σ^2^ values for models 1-3 were 0.24, 0.24, and 0.24, respectively. The intraclass correlation coefficient values for models 1-3 were 0.14, 0.14, and 0.14, respectively. The marginal *R*^2^/conditional *R*^2^ values for models 1-3 were 0.059/0.195, 0.060/0.194, and 0.068/0.200, respectively. The Akaike information criterion values for models 1-3 were 5458.745, 5457.957, and 5436.793, respectively.

^b^Includes demographic and occupational variables.

^c^Includes model 1 + restriction and seasonal factors.

^d^Includes model 2 + physical activity influences.

^f^*P*<.05.

^g^*P*<.01.

^h^*P*<.001.

^i^Not available.

^e^Italicized values denote significance.

### Midsleep Variability

Table 4 presents the factors predicting the variability of MS. Across the 3 models, the number of children (*P*=.004), snoozing behavior (*P*=.01), midsteps (*P*=.008), and IV (*P*=.001) emerged as significant factors. For each additional child, MS variability was reduced by 0.10 units (95% CI –0.16 to –0.03; *P*=.004). In all models, being a snoozer correlated with increased MS variability. Specifically, snoozers experienced a 0.17-unit increase in MS variability compared with nonsnoozers (95% CI 0.03-0.31; *P*=.01). To better understand the characteristics of snoozers, we conducted an analysis based on Mattingly et al’s study [34]. Interestingly, our results revealed that age (*P*=.02) and chronotype (*P*=.002) were significant factors in predicting snoozing behavior. The full results are detailed in Multimedia Appendix 2.

In the full model, including PA variables, midsteps also became significant. Each hour increase in midsteps was associated with a 0.02-unit decrease in MS variability (95% CI –0.04 to –0.00; *P*=.008). However, the more complex models did not show a significant improvement over the baseline model, as indicated by the LRT (model 2: *χ*^2^_2_=1.00; *P*=.60/model 3: *χ*^2^_5_=10.17; *P*=.07).

**Table 4:** Estimates of fixed effects from the linear mixed effects model predicting MS variability^a^.

Predictors	Model 1^b^				Model 2^c^				Model 3^d^			
	Estimated	CI	*P* value^e^	Estimated		CI	*P* value^e^	Estimated		CI	*P* value^e^	
Age	0.00	–0.00 to 0.01	.41	0.00		–0.00 to 0.01	.41	0.00		–0.00 to 0.01	.55	
Gender (male)	0.10	–0.03 to 0.23	.12	0.10		–0.03 to 0.23	.12	0.09		–0.04 to 0.22	.17	
Number of children	–0.09	–0.16 to –0.02	*.01^f^*	–0.09^f^		–0.16 to –0.02	*.01^f^*	–0.10		–0.16 to –0.03	*.004^g^*	
Origin (migrant background)	–0.05	–0.19 to 0.11	.49	–0.05		–0.19 to 0.11	.49	–0.05		–0.20 to 0.09	.45	
Occupation (service)	–0.12	–0.26 to 0.02	.11	–0.12		–0.25 to 0.02	.11	–0.11		–0.25 to 0.02	.10	
MEQ	0.00	–0.02 to 0.02	.96	0.00		–0.02 to 0.02	.98	0.00		–0.02 to 0.02	.94	
Snoozer (yes)	0.20	0.06 to 0.35	.*006*^g^	0.20		0.06 to 0.35	*.006^g^*	0.17		0.03 to 0.31	*.01^f^*	
Daylength	—^h^	—	—	0.00		–0.00 to 0.01	.34	0.00		–0.00 to 0.01	.26	
Stringency Index	—	—	—	0.00		–0.00 to 0.00	.94	0.00		–0.00 to 0.00	.89	
Steps (×1000)	—	—	—	—		—	—	0.00		–0.01 to 0.01	.41	
Midsteps	—	—	—	—		—	—	–0.02		–0.03 to –0.00	*.008^g^*	
Intradaily variability	—	—	—	—		—	—	–0.09		–0.21 to –0.02	09	

^a^The σ^2^ values for models 1-3 were 0.59, 0.59, and 0.59, respectively. The intraclass correlation coefficient values for models 1-3 were 0.09, 0.09, and 0.09, respectively. The marginal *R*^2^/conditional *R*^2^ values for models 1-3 were 0.034/0.120, 0.034/0.120, and 0.038/0.122, respectively. The Akaike information criterion values for models 1-3 were 8679.371, 8682.369, and 8679.197, respectively.

^b^Includes demographic and occupational variables.

^c^Includes model 1 + restriction and seasonal factors.

^d^Includes model 2 + physical activity influences.

^e^Italicized values denote significance.

^f^*P*<.05.

^g^*P*<.01.

^h^Not available.

## Discussion

### Principal Findings

In this study, we used a year-long longitudinal data set from 112 working adults and identified several significant relationships between changes in sleep over time and various factors, including restriction policies, seasonal changes, PA, and sociodemographics. We found that more stringent restrictions were associated with increased TST and delayed MS. Additionally, seasonal factors played a notable role: increased day length was linked to reduced TST and delayed MS. Changes in work arrangements, particularly the shift to remote work, directly impacted individuals based on their occupations and sleep patterns. Academic personnel, with more flexible schedules, slept less and exhibited greater variability in TST compared with service personnel, who had more structured work schedules. Additionally, individuals identified as “snoozers” had more flexible sleep schedules with greater variability in both TST and MS compared with nonsnoozers. Moreover, activity patterns played a significant role: exercising later in the day was associated with longer TST and reduced variability in both TST and MS. To contextualize our findings within the broader scope of sleep during the pandemic, the following section details our results and compares them with previous studies.

### Demographic Factors

Previous research has highlighted several epidemiological factors affecting sleep patterns, notably, age, gender, and chronotype. Consistent with previous studies, we found that older individuals tend to sleep less [43,44]. However, our findings reveal a correlation between older age and increased TST variability, which contrasts with prior results [45]. The variance in the observed correlations may be due to our study using objective sleep measures, while [45] relied on self-reported data. Additionally, we found no significant association between MS variability and age. Regarding gender differences, our study shows that males tend to have shorter and less consistent TST compared with females. While the shorter TST among males is well-documented [46,47], evidence regarding gender disparity in TST variability is inconsistent. For instance, an actigraphy study on a middle-aged cohort found that females exhibited greater TST variability than males [48]. Conversely, a survey-based study on university students [49] reported no gender differences in TST variability. Additionally, our study observes that parental duties significantly impact sleep patterns. Parents typically exhibited earlier sleep times and more consistent TST and MS than nonparents. The underlying reasons for these observations remain uncertain, but one hypothesis is that parents’ sleep/wake schedules are more stable due to the need to synchronize their sleep patterns with those of their children. While the specific relationship between parenting and sleep pattern variability has not been extensively studied, research on cohabitation suggests that living with others can influence sleep patterns by reducing variability in sleep timing and duration [50,51]. This context highlights how factors related to shared living arrangements, such as parenting, can contribute to greater sleep pattern regularity.

### Snoozing Behavior

We observed higher variability in TST and MS among individuals identified as “snoozers.” Interestingly, younger individuals and those with an evening chronotype are more likely to be “snoozers,” suggesting an interplay between age, chronotype, and snoozing habits. The natural sleep-wake patterns associated with an individual’s chronotype may influence their tendency to snooze alarms. Morning types, who wake up earlier, might not feel the need to snooze as much because their schedules align better with societal norms, in contrast to evening types.

Clinically, snoozing can be linked to prolonged sleep inertia, a state of reduced alertness upon waking [52]. Morning types (with high MEQ scores) may be less prone to snoozing and thus avoid significant sleep inertia, potentially leading to better alertness and performance. Conversely, evening types who snooze might experience greater sleep inertia, which could present additional challenges, such as managing increased work demands.

### Occupational Factors

We found that academic staff have shorter and more variable TST compared with service staff, and also exhibit greater variability in MS. The flexible and deadline-driven nature of academic schedules may contribute significantly to these irregular sleep patterns. As academics frequently adjust their schedules to meet project deadlines or prepare lectures, the dynamic nature of their workload can disrupt regular sleep patterns. Additionally, the intellectual and creative demands of academic work often extend beyond the traditional 9-5 workday, further contributing to irregular sleep schedules.

Nonetheless, it is noteworthy that increased variability in sleep patterns might impact overall health and well-being. For instance, studies using actigraphy have found that higher TST variability is associated with an increase in depressive symptoms [53,54]. These implications become even more significant in the context of the COVID-19 pandemic. The shift to remote working and learning may introduce greater flexibility for academic personnel. Although this flexibility allows for more control over schedules, it may also blur the boundaries between work and personal life, potentially leading to longer work hours and more irregular sleep patterns.

### Restriction Policies

The influence of lockdown measures during the pandemic on sleep patterns is well documented, with increased TST and later MS observed during lockdown periods [18,21,22]. Our findings further reinforce previous evidence at a more granular scale. In a more detailed analysis using the SI to measure lockdown severity, Ong et al [55] found that a higher SI was correlated with later and more variable MS. Contrary to Ong et al’s findings, our study did not find a correlation between the SI and the variability of MS. However, it is important to note the methodological differences between our studies: while Ong et al [55] conducted their correlation measurements on a monthly basis, our analysis was performed at a weekly level. These differences in granularity may account for the contrasting results.

Although not closely examined in this study, we postulate that the side effects of restriction policies might significantly impact sleep. Prolonged periods of staying at home could induce stress, potentially increasing the prevalence of insomnia [56]. Furthermore, loneliness due to self-isolation could further worsen sleep quality [57]. Despite these adverse effects, restriction policies have also had positive aspects. The shift to remote work persists, as postpandemic workplace policies increasingly encourage hybrid and remote work [58]. This change allows for greater flexibility in daily schedules, potentially leading to improved and longer sleep.

### Seasonality

Seasonal factors, such as day length, have been shown to influence sleep patterns, including sleep duration and timing [59,60]. Longer daylight hours during summer may encourage longer waking periods, while shorter days in winter can disrupt melatonin production, potentially leading to extended sleep duration. Additionally, these seasonal shifts align with changes in social schedules, such as holidays, which can further affect regular sleep routines. In southern Finland, where day length can vary by up to 13 hours between summer and winter, these influences might be more pronounced. It is possible that reduced exposure to natural daylight, due to limited mobility during the pandemic, could have altered the effect of day length on sleep.

### Physical Activity

The connection between PA and sleep has been extensively studied [61-63]. While regular PA is generally recommended for promoting good sleep, it is crucial to recognize that PA is a multifaceted behavior with various elements—such as duration, timing, and intensity—that can each influence sleep differently [61]. Therefore, investigations into the relationship between sleep and PA should consider these diverse aspects of PA.

When considering the timing of PA and its effect on sleep, our findings indicate that engaging in PA later in the day is associated with longer TST and reduced variability in both TST and MS. This supports previous research, such as a review by Youngstedt et al [63], which suggested that exercising later in the day can be beneficial for sleep. Similarly, a survey study found that engaging in light- to moderate-intensity workouts early in the evening may have beneficial effects on sleep [64]. The impact of PA’s intensity on sleep could potentially modify the effects of its timing. Sleep hygiene guidelines suggest that vigorous exercise late at night may increase arousal and subsequently impair sleep quality [65]. However, recent research challenges this convention. For instance, Myllymäki et al [66] conducted a study under controlled laboratory conditions and found that exercise performed 4 hours before bedtime did not disturb sleep. Furthermore, a review by Stutz et al [67] suggested that evening exercise does not necessarily adversely impact sleep, although exercising less than an hour before bedtime could potentially disrupt sleep.

In addition to the volume and timing of PA, we found that the fragmentation of activity rhythms, measured by IV, significantly predicted sleep patterns. Our finding of a negative association between IV and TST reinforces previous research [68], which suggests that greater fragmentation in daily PA is linked to shorter sleep duration. Additionally, the novel associations between IV and MS, as well as the variability of TST, contribute new insights into the study of activity rhythms and sleep patterns.

By leveraging longitudinal data from fitness trackers, our study highlights the potential of mHealth to offer deeper insights into behavioral health patterns, especially regarding how lifestyle changes during the pandemic have impacted sleep. This integration of mHealth approaches in sleep research exemplifies how technological advancements can enhance our understanding and interventions in public health.

### Limitations

This work has several unavoidable limitations. First, the absence of baseline data from the prepandemic period limits our study, preventing a comparison of sleep patterns and quality before and during the later stages of the pandemic. Second, the study was conducted among university staff, leading to a nonrepresentative sample that may introduce bias and result in a limited sample size. The relatively small sample size may have contributed to the wide CIs observed, indicating that the precision of our estimates could be improved. Consequently, our findings should be interpreted with caution, especially when generalizing to a broader population. Third, although we attempted to control for all known factors affecting sleep, there may still be unaddressed confounding variables. Fourth, we used consumer-grade wearables for data collection, which, despite their accessibility, may not provide the same accuracy and reliability as professional-grade equipment. Fifth, recall bias in self-reported measures is an inherent challenge. However, we addressed this issue by using validated questionnaires and conducting monthly data collection to minimize recall intervals. Finally, the study’s geographical limitation restricts the generalizability of our findings to other cultural or social contexts.

### Future Directions

One possible future research direction is to further investigate the relationship between snoozing behavior and specific demographics, such as age, to identify potential causative factors. For example, a case-control study could be conducted to compare individuals who frequently snooze with those who rarely or never do, across various age groups. This approach would enable a detailed examination of how snoozing behavior varies with age, while controlling for potential confounding variables.

### Conclusions

Our study, through a holistic approach, provided insights into the changes in sleep patterns and PA levels among working adults during the late stages of the COVID-19 pandemic. The flexible working hours during the pandemic led to corresponding flexibility in sleep patterns in certain occupations and sleep traits, particularly among individuals who self-identified as snoozers. Our findings underscore the significant impact of lifestyle habits on sleep health, particularly during unprecedented times like a global pandemic. Moving forward, it is essential to further investigate changes in sleep patterns across diverse populations. Such research will help inform workplace policies in the postpandemic era, considering the potential benefits and challenges of remote work. One notable advantage to consider is the increased amount of sleep that workers may experience, potentially enhancing overall efficiency and productivity. As we navigate the future of work, understanding the interplay between work arrangements, lifestyle choices, and sleep quality will be essential for promoting optimal well-being and performance in the workforce.

## Appendix

Multimedia Appendix 1 Additional analyses.

Multimedia Appendix 2 Supplementary Tables S1 and S2.

## Abbreviations

AIC: Akaike information criterion
IV: intradaily variability
LRT: likelihood ratio test
MEQ: Morningness-Eveningness Questionnaire
MS: midsleep
PA: physical activity
PANAS-SF: short form of the Positive and Negative Affect Schedule
PHQ-2: 2-item Patient Health Questionnaire
PSG: polysomnography
PSQI: Pittsburgh Sleep Quality Index
SI: Stringency Index
TST: total sleep time
