# Supplementary material for: Sleep During the COVID-19 Pandemic: Longitudinal Observational Study Combining Multisensor Data With Questionnaires
Source: JMIR Mhealth Uhealth. 2024 Sep 3;12:e53389. doi: 10.2196/53389 (PMC11408889; doi:10.2196/53389)
Supplement: Multimedia Appendix 1 [file mhealth_v12i1e53389_app1.docx]

## **Multimedia Appendix 1**

| **Staff** | **Academic** | | **Service** | |
| --- | --- | --- | --- | --- |
|  | **TST** | **MS** | **TST** | **MS** |
| July | 7.02 (+-1.25) | 4.50 (+-1.63) | 7.52 (+-1.13) | 4.16 (+-1.49) |
| August | 7.13 (+-1.24) | 4.16 (+-1.50) | 7.42 (+-1.11) | 3.72 (+-1.15) |
| September | 7.15 (+-1.15) | 4.06 (+-1.76) | 7.43 (+-1.09) | 3.49 (+-1.47) |
| October | 7.09 (+-1.26) | 4.09 (+-1.80) | 7.45 (+-1.13) | 3.62 (+-1.35) |
| November | 7.13 (+-1.23) | 3.91 (+-1.41) | 7.36 (+-1.06) | 3.51 (+-1.20) |
| December | 7.22 (+-1.33) | 4.28 (+-1.77) | 7.49 (+-1.21) | 3.85 (+-1.41) |
| January | 7.17 (+-1.34) | 4.30 (+-1.62) | 7.53 (+-1.12) | 3.84 (+-1.32) |
| February | 7.14 (+-1.21) | 4.04 (+-1.56) | 7.44 (+-1.10) | 3.66 (+-1.55) |
| March | 7.04 (+-1.27) | 3.87 (+-1.53) | 7.36 (+-1.07) | 3.42 (+-1.33) |
| April | 6.99 (+-1.24) | 3.90 (+-1.30) | 7.38 (+-1.12) | 3.63 (+-1.64) |
| May | 7.01 (+-1.23) | 4.12 (+-1.59) | 7.25 (+-1.10) | 3.63 (+-1.36) |

Table S1: Sleep patterns characteristic of academic and service occupation
