# Supplementary material for: Sleep During the COVID-19 Pandemic: Longitudinal Observational Study Combining Multisensor Data With Questionnaires
Source: JMIR Mhealth Uhealth. 2024 Sep 3;12:e53389. doi: 10.2196/53389 (PMC11408889; doi:10.2196/53389)
Supplement: Multimedia Appendix 2 [file mhealth_v12i1e53389_app2.docx]

## **Multimedia Appendix 2**

| **Snoozer** | **Odds ratio** | **z** | **P-value** | **95% CI** |
| --- | --- | --- | --- | --- |
| Age | 0.92 | -2.26 | *.02* | 0.86 to 0.99 |
| Gender (male) | 0.45 | -1.13 | .25 | 0.11 to 1.78 |
| Avg. steps (x1000) | 1.06 | 0.57 | .57 | 0.87 to 1.28 |
| Avg. total sleep time | 0.44 | -1.56 | .11 | 0.16 to 1.23 |
| Extraversion | 1.01 | 0.08 | .92 | 0.82 to 1.24 |
| Agreeableness | 1.13 | 0.98 | .32 | 0.89 to 1.44 |
| Conscientiousness | 0.90 | -0.82 | .41 | 0.70 to 1.16 |
| Negative Emotionality | 0.98 | -0.14 | .88 | 0.79 to 1.22 |
| Open Mindedness | 0.94 | -0.42 | .67 | 0.71 to 1.24 |
| Positive Affect | 1.06 | 0.58 | .56 | 0.87 to 1.30 |
| Negative Affect | 1.07 | 0.58 | .56 | 0.86 to 1.32 |
| PHQ-2 | 1.09 | 0.20 | .84 | 0.49 to 2.44 |
| PSQI | 1.00 | -0.002 | .99 | 0.73 to 1.37 |
| MEQ | 0.71 | -3.02 | *.002* | 0.57 to 0.89 |

Table S1: Summary of logistic regression model predicting snoozing behavior

We perform a Baron and Kenny 4-step mediation analysis [68] to discern the mediation role of chronotype on the relationship between personality and snoozing behavior. We modeled logistic regression with five personality traits as IVs, snoozing behavior as the DV, and chronotype (MEQ) as the mediator (M). At step 1, only Conscientiousness significantly predicted snoozing behavior negatively (Odds ratio = 0.79, p-value = 0.01). At step 2, a linear regression model showed that Conscientiousness was positively associated with MEQ (beta = 0.41, p < 0.001). At step 3, the logistic regression model showed that MEQ significantly predicted snoozing behavior negatively (Odds ratio = 0.79, p-value = 0.01). In a final logistic model including both MEQ and Conscientiousness, MEQ was a significant predictor of snoozing (Odds ratio = 0.86, p-value = 0.01), while Conscientiousness was not (p-value = 0.12), suggesting MEQ fully mediated this relationship. The detailed results of all steps are displayed in Table S2.

| **Path** | **Chronotype as a mediator** | | | |
| --- | --- | --- | --- | --- |
|  | Step 1  (odds ratio) | Step 2  (beta) | Step 3  (odds ratio) | Step 4  (beta) |
| C -> Sz | 0.79* |  |  | -0.15 |
| N -> Sz | 1.07 |  |  | 0.07 |
| O -> Sz | 0.92 |  |  | -0.08 |
| A -> Sz | 1.01 |  |  | 0.04 |
| E -> Sz | 0.96 |  |  | -0.06 |
| C -> Ch |  | 0.41*** |  |  |
| N -> Ch |  | -0.04 |  |  |
| O -> Ch |  | 0.08 |  |  |
| A -> Ch |  | -0.01 |  |  |
| E -> Ch |  | -0.02 |  |  |
| Ch-> Sz |  |  | 0.77 * |  |

Table S2: Mediation analysis using Baron & Kenny's method to investigate chronotype as a mediator between snoozing behavior and personality traits. Step 1 = IV -> DV; Step 2 = IV -> M; Step 3 = M -> DV; Step 4 = (IV + M) ->DV.

IV = independent variable; DV = dependent variable; M = mediator.

Sz = Snoozer; Ch = Chronotype (MEQ); C = Conscientiousness; N = Neuroticisim; O = Openness; A = Agreeableness; E = Extraversion.

*p<0.05. ** p<0.01. *** p<0.001.
